# Specific expression profile and prognostic significance of peroxiredoxins in grade II-IV astrocytic brain tumors

**DOI:** 10.1186/1471-2407-10-104

**Published:** 2010-03-22

**Authors:** Sally Järvelä, Immo Rantala, Alejandra Rodriguez, Heini Kallio, Seppo Parkkila, Vuokko L Kinnula, Ylermi Soini, Hannu Haapasalo

**Affiliations:** 1 Department of Pathology, Centre for Laboratory Medicine, Tampere University Hospital, Tampere, Finland; 2 Institute of Medical Technology and School of Medicine, University of Tampere and Tampere University Hospital; 3 Department of Medicine, Division of Pulmonary Medicine, Helsinki University Hospital, Helsinki, Finland; 4 Department of Pathology, University of Kuopio, Kuopio, Finland

## Abstract

**Background:**

Peroxiredoxins (Prxs) have recently been suggested to have a role in tumorigenesis.

**Methods:**

We studied the expression of Prx I-VI and their relationship to patient survival in 383 grade II-IV diffuse astrocytic brain tumors.

**Results:**

Prx I positivity was found in 68%, Prx II in 84%, Prx III in 90%, Prx IV in 5%, Prx V in 4% and Prx VI in 47% of the tumors. Prx I and Prx II expression decreased significantly with increasing malignancy grade (p < 0.001 and p < 0.001). Patients with Prx I or Prx II positive tumors were significantly younger than the average age of all the patients (p = 0.014 and p = 0.005). A lower proliferation rate was associated with Prx I and Prx VI positive tumors (p = 0.019 and p = 0.033), and a lower apoptotic rate was found within Prx I and Prx II positive tumors (p < 0.001 and p = 0.007). Patients with Prx I and Prx II positive tumors had a significantly better survival rate than their Prx-negative counterparts (p = 0.0052 and p = 0.0002).

**Conclusion:**

The expression of Prx I and Prx II correlates with astrocytic tumor features, such as grade and patient age and proliferation activity (Prx I), and accordingly with patient survival.

## Background

Diffusely infiltrating astrocytomas of grades II - IV are the most frequent primary brain tumors accounting for more than 30% of central nervous system tumors. Although they may develop at any age, the majority of astrocytomas manifest clinically in adults. Grade II astrocytomas represent the least malignant cases, whereas grades III (anaplastic astrocytomas) and IV (glioblastomas or GBM) are highly malignant and have a poor prognosis. GBM is one of the most aggressive human neoplasms [1].

Peroxiredoxins (Prx) are a family of thiol-specific antioxidant enzymes (AOE) that have a role in cellular antioxidant defence by breaking down H_2_0_2 _[2,3]. Prxs are also thought to be associated with cell proliferation, apoptosis, differentiation, gene expression and resistance to radiation or chemotherapy [4-8]. Microglial cells and astrocytes have been shown weakly or moderately immunoreactive for Prx I and Prx VI, and oligodendrocytes for Prx I and Prx IV. Neurons do not seem to have immunoreactivity for Prx I and Prx VI, while all other Prxs are expressed weakly to moderately [9].

Studies on human carcinomas of the thyroid gland, pleural mesothelioma, oral and lung cancer, prostate cancer and breast carcinoma suggest that Prxs may have a role both in tumor progression and in drug resistance [10-16]. It is also possible that Prx proteins have a direct influence on tumorigenesis.

Overall the importance of the Prx family of proteins in human tumors remains unclear. They have not been earlier investigated in grade II-IV diffuse astrocytomas. This prompted us to investigate the expression of all six peroxiredoxins in 383 grade II-IV astrocytic brain tumors and to examine how they correlate with tumor features and patient survival.

## Methods

### Tumor samples

Histological samples of astrocytic tumors were obtained from 383 patients who underwent surgery at Tampere University Hospital, Tampere, Finland in 1983-1997. 299 samples were from primary tumors and 84 from recurrent ones. Their distribution by malignancy grade is shown in Table 1.

**Table 1:** Description of the astrocytic tumors: Association of Prx expression with tumor grade in the total material.

Tumor grade	Primary tumors	Recurrent tumors	Total	PrxI +/-	PrxII +/-	PrxIII +/-	PrxIV +/-	PrxV +/-	PrxVI +/-
Grade II	48	14	62	17/30	34/12	25/22	-/47	-/47	5/40
Grade III	35	19	54	14/28	28/15	27/16	-/44	-/43	2/39
Grade IV	216	51	267	33/184	98/120	126/87	1/209	-/211	26/189

Total	299	84	383	64/242	160/147	178/125	1/300	-/301	33/268
				*	**	n.s.	n.s.	n.s.	n.s.

Negative or weak staining was considered as Prx-negative expression (Prx-) and moderate or strong staining as Prx-positive expression (Prx+). * p < 0.001, chi-square test

** p < 0.001, chi-square test

The median age of the patients at the time of operation was 48.9 years (mean 48.8, SD 15.0) and the male-female ratio was 1.3:1. On average, patients with recurrent tumors were nine years younger than those with primary tumors. All the patients underwent neurosurgical operation with the intention of gross radical tumor resection. The patients with primary tumors that were included to this study did not receive any anticancer medication prior to the operation. The tumor samples were fixed in 4% phosphate-buffered formaldehyde and embedded in paraffin. Paraffin sections were stained with haematoxylin-eosin. Histopathologic typing and grading was carried out according to WHO criteria [1]. Histologically representative tumor regions were selected by a neuropathologist (HH) and the samples from these areas were applicated in tissue microarray blocks using a custom-built instrument (Beecher Instruments, Silver Spring, MD, USA). The diameter of the tissue cores in the microarray blocks was 600 μm. When assessing the role of malignancy grade, grade II and III astrocytomas were considered as one group and compared to glioblastomas (grade IV astrocytomas).

Immunohistology. The immunohistochemical (IHC) procedure was as follows. Four-micron thick sections were cut from the microarray blocks. The sections were then deparaffinised in xylene and rehydrated in descending ethanol series. For antigen retrieval, the sections were incubated in 10 mM citrate buffer (pH 6.0) in a microwave oven, 2 min at 850 W followed by 8 min at 350 W. Endogenous peroxidase activity was blocked by incubation in 0.1% hydrogen peroxide in absolute methanol for 10 min. The polyclonal anti-Prx-antibodies were a gift from Dr Kang (Center for Cell Signalling Research and Division of Molecular Sciences, Ewha Womans University, Seoul, Korea). The dilution for the primary antibodies were 1:1500 for Prx I, 1:1000 for Prx II, 1:500 for Prx III, 1:1000 for Prx IV and 1:2000 for Prxs V and VI. For positive controls we used malignant mesothelioma samples previously known to be positive [14]. Negative control staining was carried out by substituting PBS and serum isotype controls (Zymed Laboratories Inc.) for the primary antibodies.

The primary antibodies for Prx I-VI were revealed using the Histostain-Plus Kit (Zymed Laboratories Inc, South San Francisco, CA) as described previously [17].

The immunohistochemical staining results were evaluated for each immunohistochemical target during one session on a multiheaded microscope by three observers semiquantitatively by dividing the AOE, PRX or CA IX staining reaction into four categories based on the reactivity of the staining taking equally into account both the intensity and extent of the staining: 0 = no immunostaining present; 1 = weak immunostaining, < 10% of the tumor tissue immunostained; 2 = moderate immunostaining, 10-50% of the tumor tissue immunostained; 3 = strong immunostaining present, > 50% of the tumor tissue immunostained. When Prx negativity or positivity were under comparison, the four categories were divided into two groups: the Prx negative group (Prx-) contained negatively and weakly stained tumors and the Prx positive group (Prx+) contained tumors showing moderate or strong immunostaining.

### Cell proliferation

**Figure 1:**
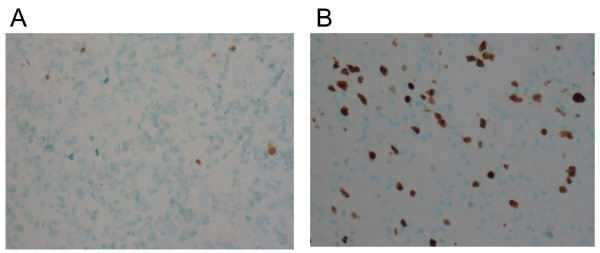
**Apoptotic rate marker TUNEL-labelling (A) and proliferation index marker MIB-1 (B) in astrocytic brain tumor samples**.

For analysis of cell proliferation, a mouse monoclonal antibody MIB-1 recognising the Ki-67 antigen was used (Immunotech, S.A. Marseille, France) (dilution 1:40). After immunostaining, the tissue sections were counterstained with methyl green. Proliferative activity was reported as the percentage of immunopositive nuclei. Analysis of all tumor cells was done with an image analysis system (CAS-200 TM Software, Becton Dickinson & Co., USA) as described previously [18] (Figure 1.).

### Apoptotic rate

The activity of apoptosis was determined by TUNEL-labelling. Deparaffinised tumor microarray tissue sections were first digested with proteinase K (20 μg/ml) for 15 min. Apoptotic cells were demonstrated using ApopTag In Situ Apoptosis Detection Kit (Oncor, Inc., Gaithersburg, MD, USA) according to the manufacturer's instructions. In the terminal deoxynucleotidyl transferase nick end labelling method, the recommended concentration was reduced by 8-fold. Direct immunoperoxidase detection of digoxigenin labelled dUTP was followed by counterstaining in methyl green [19] (Figure 1.).

### Statistical analysis

The tests used for statistical analysis were all part of SPSS 11.0 (SPSS Inc., Chicago, Illinois) for Windows software. Chi-square tests-, t-tests, Mann-Whitney tests were used, as well as multivariate analysis of variance (ANOVA). The log-rank test and Cox multivariate analysis were used for analysis of prognostic factors. The significance level was set at *p *< 0.05.

### Ethics

The study design was approved by the Ethics committee of Tampere University Hospital and the National Authority for Medicolegal Affairs.

## Results

### Expression

**Figure 2:**
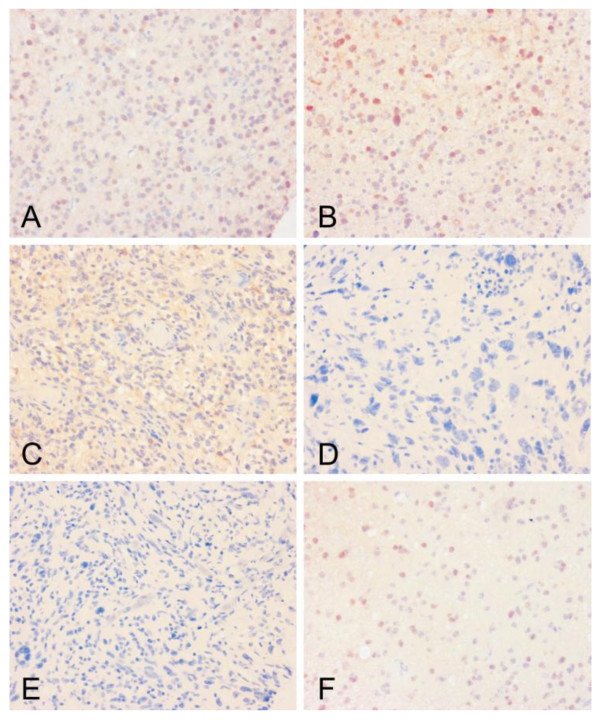
**Expression of peroxiredoxins I-VI in astrocytic brain tumors**. Positive expression is seen as an immunohistological red staining in nucleus of the astrocytoma cells (Prx I, A; Prx II, B and Prx VI, F) and in the cytoplasma (Prx III, C). Prx IV (D) and Prx V (E) stainings are negative. The figure panel consist astrocytic brain tumors of various grades (grade II, B, F; grade III A, grade IV (gbm) C, D, E).

The expression of Prxs I-VI was analyzed in 299 primary and 84 recurrent grade II-IV astrocytic tumors. The distribution of immunopositivity for each Prx is shown in Table 2. Examples of immunopositive samples of Prx I, II, III and VI and immunonegative samples of Prx IV and V are shown in Figure 2A-F.

**Table 2:** Distribution of immunostaining intensity of peroxiredoxins in astrocytic tumor samples.

Intensity of immunostaining/peroxiredoxin	Negative	Weak	Moderate	Strong	Prx- group/ Prx+ group
Prx I	32%	47%	18%	3%	79%/21%
Prx II	16%	32%	30%	22%	48%/52%
Prx III	10%	31%	39%	20%	41%/59%
Prx IV	95%	5%	< 1%	-	100%/-
Prx V	96%	4%	-	-	100%/-
Prx VI	53%	36%	10%	1%	89%/11%

For the analyses, the four categories are further divided into two groups: The Prx negative group (Prx-) contains negatively and weakly stained tumors and Prx positive group (Prx+) contains tumors showing moderate or strong immunostaining.

**Figure 3:**
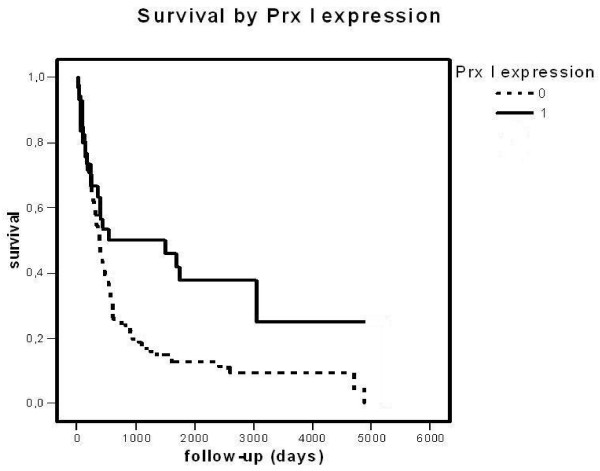
**Survival by peroxiredoxin I in primary astrocytic brain tumors**. Tumors were divided in two groups, Prx+ and Prx-. The difference between the groups is significant (p = 0.0052, log-rank test).

The immunostaining intensity of each Prx in different grades is shown in Figure 3. Higher grade tumors showed a significant decrease in immunoreactivity for Prx I and Prx II (p < 0.001 and p < 0.001, chi-square tests). The distribution of malignancy grade in Prx+ and Prx- groups is shown in Table 1.

Prx I was positive in 66% of primary tumors and in 75% of recurrent tumors (p = 0.008, chi-square test). Similarly, Prx VI was positive in 45% of primary tumors and in 54% recurrent tumors (p = 0.01, chi-square test). Patients with Prx I+ and Prx II+ primary tumors were significantly younger than their Prx- counterparts (p = 0.014 and p = 0.005, t-test) (Table 3).

**Table 3:** Expression of Prx, association with patient and tumor characteristics.

Characteristic	PrxI +/-	PrxII +/-	PrxIII +/-	PrxIV +/-	PrxV +/-	PrxVI +/-
PATIENT						
Age						
Mean	45.0/52.5	47.9/54.6	51.8/50.2	*#*/50.9	*#*/51.1	52.9/51.0
Median	39.8/54.4	45.6/56.3	54.1/51.9	*#*/53.8	*#*/53.9	56.8/53.5
	p = 0.014*	p = 0.005*	n.s.			n.s.
Sex						
Male	37/118	86/70	101/56	1/154	*#*/156	13/139
Female	21/93	55/59	59/51	-/110	*#*/110	12/102
	n.s.	n.s.	n.s.	n.s.		n.s.
Recurrences						
None	42/193	117/118	136/96	1/229	*#*/231	29/201
≥ 1	22/49	43/49	42/29	-/71	*#*/70	4/67
	p = 0.015**	n.s.	n.s.	n.s.		n.s.
Tumor proliferation (MIB-1)						
Mean						
SD	13.8/16.9	16.2/16.5	17.5/15.2	*#*/16.5	*#*/16.4	11.1/16.8
Median	23.7/21.6	24.5/18.9	22.7/22.3	*#*/22.2	*#*/22.2	18.1/22.0
	5.0/10.4	8.2/12.2	10.6/7.1	*#*/9.4	*#*/9.4	5.0/10.0
	p = 0.019***	n.s.	p = 0.056****			p = 0.033***
Tumor Apoptosis						
Mean	5.5/10.4	7.6/11.6	8.7/10.7	*#*/9.6	*#*/9.6	8.9/9.4
SD	12.1/15.9	12.7/17.9	13.5/18.0	*#*/15.2	*#*/15.2	14.2/15.6
Median	3.6/7.1	3.6/7.1	3.6/3.6	*#*/3.6	*#*/3.6	3.6/3.6
	p < 0.001***	p = 0.007***	n.s.			n.s.

* T-test, only primary tumors were included

** Chi-square test

*** Mann-Whitney test

**** Near-significant p-value by Mann-Whitney test

*# *No cases in prx-positive group, analysis of significance not possible

We also studied the intercorrelation of Prxs. Prx I positivity correlated significantly with Prx II and Prx III positivity (p < 0.001 and p = 0.014, chi-square tests), and Prx II positivity correlated significantly with Prx III and Prx VI positivity (p = 0.005 and p = 0.031, chi-square tests): these Prx+ tumors increased considerably when the other Prxs expressed positivity.

### Proliferation and apoptosis

In the total material the mean MIB-1 proliferation index was 16.0 (SD 20.88) and median 9.7%. Proliferation activity was compared between Prx+ and Prx- groups. Prx I+ and Prx VI+ tumors had a significantly lower proliferation rate than their IHC negative counterparts (p = 0.019 and p = 0.033, Mann-Whitney test). In contrast, Prx III+ tumors had a marginally higher proliferation rate than Prx III- tumors (p = 0.056, Mann-Whitney test). The results are compiled in Table 3.

The mean for the TUNEL labelling index in the total material was 9.6 (SD 15.2) and median 3.6. The tumor apoptotic rate was decreased in Prx I+ and Prx II+ tumors (p < 0.001 and p = 0.007, Mann-Whitney tests) (Table 3).

When the malignancy grades were divided into two groups (grades II and III vs. grade IV) and analyzed separately, the proliferation index differed significantly in Prx III+ and Prx VI+ lower grade (II and III) astrocytomas compared to their Prx III- and Prx VI- counterparts. The mean for Prx III+ tumors was 14.7 (median 4.1, SD 26.2); in Prx- counterparts it was 8.4 (median 3.0, SD 17.8) (p = 0.043, Mann-Whitney test). The mean proliferation rate in Prx VI+ cases was 1.6 (median 2.2, SD 1.6) and in Prx VI- counterparts 12.1 (median 4.1, SD 22.4) (p = 0.016, Mann-Whitney test). There was no statistically significant difference in the proliferation index when only GBMs were included in the analysis, nor in the case of other Prxs.

The apoptotic rate differed significantly only in the case of Prx I and in GBMs, when the lower grade astrocytomas and GBMs were analyzed separately. The mean apoptotic rate for Prx I+ GBMs was 8.8 (median 3.6, SD 17.6) and for Prx I- GBMs 12.8 (median 7.1, SD 17.8) (p = 0.009, Mann-Whitney test).

### Survival

Only the primary cases were included in the survival analysis.

**Figure 4:**
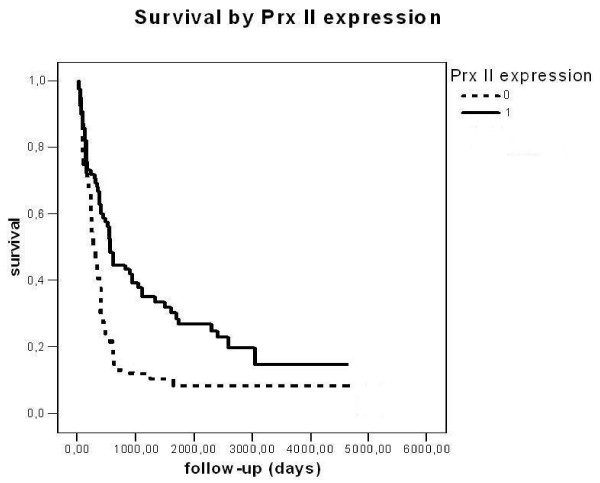
**Survival by peroxiredoxin II in primary astrocytic brain tumors**. Tumors were divided in two groups, Prx+ and Prx-. The difference between the groups is significant (p = 0.0002, log-rank test).

When Prx+ and Prx- tumors were compared, patients with Prx I+ and Prx II+ tumors showed better survival rate than their negative counterparts, Prx I+ nearly significantly and Prx II+ significantly (p = 0.0052 and p = 0.0002, log-rank tests) (Figures 3 and 4). Other peroxiredoxins did not reach statistical significance.

A further survival analysis was carried out for the patients with glioblastomas (N = 216) or lower grade (grade II and III) astrocytoma (N = 83). The mean follow-up for GBMs was 433 days (16.2% alive after mean follow-up) and for lower grade astrocytomas 1645 days (30.1% alive after mean follow-up). In this comparison, Prx I had a favourable effect on prognosis in lower grade astrocytomas (p = 0.0072, log-rank test). In GBMs no corresponding statistical significance was found in patient survival.

Proliferation index, patient age (in days), tumor grade, sex and Prxs were included in the Cox multivariate analysis. Only patient age and tumor grade had independent prognostic significance (p < 0.001, Odds ratio (OR) = 2.207 and p < 0.001, OR = 1.874).

## Discussion

We investigated the role of Prxs in a large series of diffusely infiltrating (grade II-IV) astrocytic brain tumors. Prx I and Prx II in particular showed interesting associations with tumor features and patient survival. The majority of the astrocytic tumors were positive for Prx I, Prx II and Prx III, while Prxs IV-VI showed lower expression. The proportion of Prx I and Prx II immunopositive tumors decreased with increasing malignancy grade. However, Prx I was more frequent in recurrent tumors than in primary ones. Prx I and Prx II were associated with patient prognosis, patients with positive tumors living significantly longer than their negative counterparts.

Our results suggest that Prxs have a role in the behavior of astrocytic brain tumors, a role that seems to go beyond that of an antioxidant enzyme. This notion is underscored by their very low H_2_O_2 _catalysing activity and their tendency to be inactivated during the H_2_O_2 _catalytic process, even at very low concentrations of H_2_O_2 _[20,21]. We are only beginning to understand the many and varied functions of Prxs. Their versatility is likely to be comparable to that of thioredoxins [22]. Prxs act as reductases for alkyl hydroperoxide, H_2_O_2 _and peroxynitrite, and they seem to regulate peroxide-mediated signaling cascades as well as NF-κB activation [21,23,24]. Although classified as peroxidases, they already are known to regulate mitogen-activated protein kinase activity [25], and they are associated with cell proliferation and growth control, differentiation, immune responses, apoptosis and tumorigenesis, pathogenesis of neurodegenerative disorders and resistance to radiation and/or drug therapy [7,26-29]. They are needed for Myc-mediated transformation and apoptosis [5,30] and they may have a role in tumorgenesis, as they are aberrantly overexpressed in several cancers [10-16]. The involvement of Prxs in these many cell functions is important because NF-κB, cell proliferation, differentiation and apoptotic activity have been found to be characteristically altered in astrocytomas and to have an effect on tumor behavior or even on prognosis [31-33].

Genes of Prxs can be induced by cellular stresses, including hypoxia and rapid growth, which are typical of aggressive tumors such as astrocytomas and particularly GBMs. This could explain the association between different antioxidants in rapidly growing malignancies: changes in the cellular environment affect the expression of more than one antioxidant enzyme at the same time. The same phenomenon may be caused by advanced treatments of the cancer: radiation or cytotoxic drugs, which often generate reactive oxygen metabolites (e.g. cisplatin), cause oxidant stress at the cellular level with consequent effects on the antioxidant machinery; in some cases this may contribute to increased resistance to such therapies. Resistance to anticancer drugs and radiation therapy has already been reported by Park and colleagues and Chung and colleagues in the case of Prx II in gastric and head and neck cancer cell lines [7,8].

In our astrocytoma material we found several correlations of Prx I with tumor features and even with patient survival. Patients with Prx I immunopositive tumors were younger and they also lived longer. Furthermore, compared to patients whose tumors did not express Prx I, their tumors showed a lower proliferation rate and on the other hand decreased apoptotic activity. However, this seemingly contradictory result in prognosis and apoptotic rate makes perfect sense when the tumors are divided into two groups not only on the basis of their Prx I status but also their histopathology. The beneficial connection between Prx I positivity and prognosis was significant mainly in grade II and III astrocytomas, whereas no such statistically significant association was seen in GBMs. At the same time the decreasing effect of Prx I positivity on the apoptotic rate was significant only in glioblastomas when the histopathology of the tumor was taken into account. Even though it would be favorable for the prognosis to have a higher apoptotic rate, GBMs are known to be maximally aggressive and rapidly growing tumors, so the effect on apoptosis would need to be very high in order to be reflected in the prognosis. In the case of grade II and III astrocytic tumors, the effect of Prx I on the prognosis do not seem to correlate with apoptosis. Prx I did correlate with tumor malignancy grade, but it was not an independent prognostic factor. Nevertheless, the association of Prx I with malignancy grade and proliferation suggests that Prx I, partly via its association with these factors, has a role in the pathogenesis of grade II-III astrocytomas.

In normal mammalian brain immunoreactivity for Prx II has been found principally in the cytosol of most neurons of grey matter [9,34,35]. It has also been found in the nuclei of medial habenular neurons [9]. These are involved in many important and even vital biological functions, and the nuclear localisation of Prx II indicates that it may have a role that goes beyond that of an antioxidative protein in normal neural tissue. Furthermore, Prx II seems to have several important independent functions in various other human cells. For example, lack of its expression is known to damage severely the normal function of erythrocytes [35]. In the pathogenesis of neurodegenerative disorders, including Alzheimer's disease, Pick's disease and Down syndrome [27], the expression of Prx II is known to be aberrant. Elevated Prx II expression has also been reported at least in lung carcinoma and pleural mesothelioma [13,14], and it is known to affect radiation sensitivity and drug resistance [7,8].

In our material Prx II was associated with the tumor apoptotic rate and the grade of malignancy. Like in the case of Prx I, univariate analysis showed that Prx II had a significant effect on prognosis. However, the median age of the patients with Prx I and Prx II-positive tumors was significantly less than of their Prx-negative counterparts and thus explains part of this favorable effect to prognosis.

Prx II positivity was found in the vast majority of tumors (in 84%), and Prx II seemed to be expressed to a greater extent that has been reported previously in normal brain tissue, for example in the study of Sarafian [34]. In that study Prx II was found exclusively in neurons and hardly at all in glial cells. The upregulation of Prx II could thus be a sign of an ultimate attempt of growing astrocytoma cells to react to the cellular stress and to resulting hypoxia/redox imbalance in the tissue.

Prx III was expressed in 90% of astrocytomas being the most often expressed peroxiredoxin in our study. In grade II-III astrocytomas it was associated with a higher proliferation rate, but there was no straightforward association with apoptosis. In normal brain tissue its immunoreactivity is concentrated to neurons especially in the hippocampal area, where it has a protective role against excitotoxic injuries [9,36]. The uneven concentration of Prx III immunoreactivity in hippocampus results not only from its function, but derives from its subcellular localisation in mitochondria. It has been suggested that Prx III is a critical regulator of apoptotic signalling by virtue of its regulatory effect on the abundance of mitochondrial H_2_O_2 _[37]. In our material this association was not evident.

Only a few cases were immunoreactive for Prx IV and Prx V in our material. In normal mammalian brain tissue, moderate Prx IV immunoreactivity has been reported in the cytoplasm of neurons and strong, mainly nuclear positivity in oligodendrocytes [9]. This could mean that Prx IV has at least partially different roles in these cells. Based on our results, it seems that Prx IV is not abundant in neoplastic astrocytes. This is also the case with Prx V, which is found in normal neural tissue in mouse brain [9] and is known to have a protective role against excitotoxic brain lesions in newborn mice [38].

Prx VI immunoreactivity has been previously found in normal mouse astrocytes and oligodendrocytes. Interestingly, the Prx VI protein has been shown to be expressed in the nuclei of astrocytes and oligodendrocytes, although Prx VI is known to be cytosolic in other mammalian cells (e.g. [9,21]). This could mean that Prx VI carries out a different function in the neural system than in other organs, such as the lungs and kidneys, where it has been previously reported to be found in the cytosol [39]. In our material almost half of the tumors showed immunoreactivity for Prx VI, most of them faintly or moderately. In recurrent tumors the proportion of immunopositive tumors increased significantly. In addition, Prx VI positive tumors showed a lower proliferation rate than negative ones, especially in grade II and III gliomas.

The biological functions and regulation of Prxs seem to be quite complex. This became more evident in a reverse transcription polymerase chain reaction analysis in which we studied PrxI, Prx II and Prx III mRNA levels in six frozen tumor specimens. Like in many other cases noticed in our laboratory, the mRNA and protein expression levels do not always correlate well with each other in biological models. This phenomenon was again confirmed here since we found no solid correlation between the studied Prx mRNA and protein expression levels. Although the sample size in the mRNA analysis was too small to make any final conclusions, we consider the semiquantitative assessment of immunostaining reactivity a more reliable marker for the actual protein expression level.

To our knowledge, there is only few studies, and with limited number of cases, concerning the role of Prxs in astrocytomas. The study of Odreman [40] showed partly contradictory data to our results. In their study 10 fibrillary astrocytomas of grade II-III and 10 glioblastomas were analyzed for their Prx I and Prx VI protein pattern by 2D electrophoresis. They used Western and immunohistochemical analysis to confirm the differential expression of the identified proteins. The expression of both Prx I and Prx VI was found to be significantly stronger in high-grade tumors compared to low-grade tumors [40]. In our considerably larger study of 383 astrocytomas Prx I was more frequently expressed in grade II and III tumors when compared to GBMs, but Prx VI expression did not have any statistically significant difference between these grades.

## Conclusions

In conclusion, our results suggest that the family of Prxs has an important role in the biology of astrocytomas. Especially Prx I and Prx II expression seemed to have correlation with the malignancy of astrocytic cells. They correlated with cell proliferation, apoptosis and malignancy grade, the main features of tumors for diagnostic and prognostic purposes. They also had an association with patient age and in univariate analysis with patient survival. Even though the correlation with prognosis may be explained by the association with other features (age, grade), Prx I and Prx II seemed to be important in tumorigenesis of astrocytic tumors.

## Competing interests

The authors declare that they have no competing interests.

## Authors' contributions

SJ participated in the study design, analyzing of the immunoassays and performed the statistical analysis and drafted the manuscript; IR participated in design of the study and revised the manuscript critically for important intellectual content; AR, HK and SP prepared the mRNA analysis and SP revised the manuscript critically for important intellectual content; VK participated on study designing and revised the manuscript critically for important intellectual content; YS participated in study design, prepared the immunoassays, participated on analyzing them and revised manuscript critically; HH participated in the study design, participated on analyzing immunoassays and all the authors have given final approval of the version to be published.

## Pre-publication history

The pre-publication history for this paper can be accessed here:

http://www.biomedcentral.com/1471-2407/10/104/prepub

## References

[B1] Kleihues P Cavanee WK Kleihues P, Cavanee WK In the book Pathology and genetics Tumors of the nervous system World Health Organization classification of tumors 2000 Lyon: International Agency for Research on Cancer 10 21 and 29-44

[B2] Wong CM Chun AC Kok KH Zhou Y Fung PC Kung HF Jeang KT Jin DY Characterization of human and mouse peroxiredoxin IV: evidence for inhibition by Prx-IV of epidermal growth factor- and p53-induced reactive oxygen species Antioxid Redox Signal 2000 2 3 507 518 10.1089/15230860050192288 11229364

[B3] Kang SW Chae HZ Seo MS Kim K Baines IC Rhee SG Mammalian peroxiredoxin isoforms can reduce hydrogen peroxide generated in response to growth factors and tumor necrosis factor-alpha J Biol Chem 1998 13;273 11 6297 6302 10.1074/jbc.273.11.6297 9497357

[B4] Chae HZ Kim HJ Kang SW Rhee SG Characterization of three isoforms of mammalian peroxiredoxin that reduce peroxides in the presence of thioredoxin Diabetes Res Clin Pract 1999 45 2-3 101 112 10.1016/S0168-8227(99)00037-6 10588361

[B5] Kim H Lee TH Park ES Suh JM Park SJ Chung HK Kwon OY Kim YK Ro HK Shong M Role of peroxiredoxins in regulating intracellular hydrogen peroxide and hydrogen peroxide-induced apoptosis in thyroid cells J Biol Chem 2000 16;275 24 18266 18270 10.1074/jbc.275.24.18266 10849441

[B6] Sasagawa I Matsuki S Suzuki Y Iuchi Y Tohya K Kimura M Nakada T Fujii J Possible involvement of the membrane-bound form of peroxiredoxin 4 in acrosome formation during spermiogenesis of rats Eur J Biochem 2001 268 10 3053 3061 10.1046/j.1432-1327.2001.02200.x 11358524

[B7] Park SH Chung YM Lee YS Kim HJ Kim JS Chae HZ Yoo YD Antisense of human peroxiredoxin II enhances radiation-induced cell death Clin Cancer Res 2000 6 12 4915 4920 11156252

[B8] Chung YM Yoo YD Park JK Kim YT Kim HJ Increased expression of peroxiredoxin II confers resistance to cisplatin Anticancer Res 2001 21 2A 1129 1133 11396151

[B9] Jin MH Lee YH Kim JM Sun HN Moon EY Shong MH Kim SU Lee SH Lee TH Yu DY Lee DS Characterization of neural cell types expressing peroxiredoxins in mouse brain Neurosci Lett 2005 24;381 3 252 257 10.1016/j.neulet.2005.02.048 15896479

[B10] Yanagawa T Ishikawa T Ishii T Tabuchi K Iwasa S Bannai S Omura K Suzuki H Yoshida H Peroxiredoxin I expression in human thyroid tumors Cancer Lett 1999 18;145 1-2 127 132 10.1016/S0304-3835(99)00243-8 10530780

[B11] Yanagawa T Iwasa S Ishii T Tabuchi K Yasa H Onizawa K Omura K Harada H Suzuki H Yoshida H Peroxiredoxin I expression in oral cancer: a potential new tumor marker Cancer Lett 2000 1;156 1 27 35 10.1016/S0304-3835(00)00434-1 10840156

[B12] Noh DY Ahn SJ Lee RA Kim SW Park IA Chae HZ Overexpression of peroxiredoxin in human breast cancer Anticancer Res 2001 21 3B 2085 2090 11497302

[B13] Lehtonen ST Svensk AM Soini Y Paakko P Hirvikoski P Kang SW Saily M Kinnula VL Peroxiredoxins, a novel protein family in lung cancer Int J Cancer 2004 10;111 4 514 521 10.1002/ijc.20294 15239128

[B14] Kinnula VL Lehtonen S Sormunen R Kaarteenaho-Wiik R Kang SW Rhee SG Soini Y Overexpression of peroxiredoxins I, II, III, V and VI in malignant mesothelioma J Pathol 2002 196 3 316 323 10.1002/path.1042 11857495

[B15] Shen C Nathan C Nonredundant antioxidant defense by multiple two-cysteine peroxiredoxins in human prostate cancer cells Mol Med 2002 8 2 95 102 12080185 PMC2039972

[B16] Karihtala P Mantyniemi A Kang SW Kinnula VL Soini Y Peroxiredoxins in breast carcinoma Clin Cancer Res 2003 15;9 9 3418 3424 12960131

[B17] Haapasalo H Kylaniemi M Paunu N Kinnula VL Soini Y Expression of antioxidant enzymes in astrocytic brain tumors Brain Pathol 2003 13 2 155 164 12744469 10.1111/j.1750-3639.2003.tb00015.x PMC8096025

[B18] Sallinen PK Haapasalo HK Visakorpi T Helen PT Rantala IS Isola JJ Helin HJ Prognostication of astrocytomas patient survival by Ki-67 (MIB-1), PCNA and S-phase fraction using archival paraffin-embedded samples J Pathol 1994 174 4 275 282 10.1002/path.1711740407 7884589

[B19] Miettinen HE Paunu N Rantala I Kalimo H Paljärvi L Helin H Haapasalo H Cell cycle regulators (p51, p53, pRb) in oligodendrocytic tumors: a study by novel tumor microarray technique J Neurooncol 2001 55 1 29 37 10.1023/A:1012961918848 11804280

[B20] Woo HA Chae HZ Hwang SC Yang KS Kang SW Kim K Rhee SG Reversing the inactivation of peroxiredoxins caused by cysteine sulfinic acid formation Science 2003 25;300 5619 653 656 10.1126/science.1080273 12714748

[B21] Wood ZA Poole LB Karplus PA Peroxiredoxin evolution and the regulation of hydrogen peroxide signaling Science 2003 25;300 5619 650 653 10.1126/science.1080405 12714747

[B22] Powis G Montfort WR Properties and biological activities of thioredoxins Annu Rev Biophys Biomol Struct 2001 30 421 455 10.1146/annurev.biophys.30.1.421 11441809

[B23] Hofmann B Hecht HJ Flohe L Peroxiredoxins Biol Chem 2002 383 3-4 347 364 10.1515/BC.2002.040 12033427

[B24] Bryk R Griffin P Nathan C Peroxynitrite reductase activity of bacterial peroxiredoxins Nature 2000 14;407 6801 211 215 10.1038/35025109 11001062

[B25] Veal EA Findlay VJ Day AM Bozonet SM Evans JM Quinn J Morgan BA A 2-Cys peroxiredoxins regulates peroxide-induced oxidation and activation of a stress-activated MAP kinase Moll Cell 2004 2;15 1 129 139 10.1016/j.molcel.2004.06.021 15225554

[B26] Hirotsu S Abe Y Okada K Nagahara N Hori H Nishino T Hakoshima T Crystal structure of a multifunctional 2-Cys peroxiredoxins heme-binding protein 23 kDa/proliferation-associated gene product Proc Natl Acad Sci USA 1999 26;96 22 12333 12338 10.1073/pnas.96.22.12333 PMC22917 10535922

[B27] Krapfenbauer K Engidawork E Cairns N Fountoulakis M Lubec G Aberrant expression of peroxiredoxin subtypes in neurodegenerative disorders Brain Res 2003 28;967 1-2 152 160 10.1016/S0006-8993(02)04243-9 12650976

[B28] Chen WC McBride WH Iwamoto KS Barber CL Wang CC Oh YT Liao YP Hong JH de Vellis J Shau H Induction of radioprotective peroxiredoxin-I by ionizing irradiation J Neurosci Res 2002 15;70 6 794 798 10.1002/jnr.10435 12444601

[B29] Nonn L Berggren M Powis G Increased expression of mitochondrial peroxiredoxin-3 (thioredoxin peroxidase-2) protects cancer cells against hypoxia and drug-induced hydrogen peroxide-dependent apoptosis Mol Cancer Res 2003 1 9 682 689 12861054

[B30] Wonsey DR Zeller KI Dang CV The c-Myc targeted gene PRDX3 is required for mitochondrial homeostasis and neoplastic transformation Proc Natl Acad Sci USA 2002 14;99 10 6649 6654 10.1073/pnas.102523299 PMC124457 12011429

[B31] Robe PA Bentires-Alj M Bonif M Rogister B Deprez M Haddada H Khac MT Jolois O Erkmen K Merville MP Black PM Bours V In vitro and in vivo activity of the nuclear factor-kappaB inhibitor sulfasalazine in human glioblastomas Clin Cancer Res 2004 15;10 16 5595 5603 10.1158/1078-0432.CCR-03-0392 15328202

[B32] Heesters MA Koudstaal J Go KG Molenaar WM Analysis of proliferation and apoptosis in brain gliomas: prognostic and clinical value J Neurooncol 1999 44 3 255 266 10.1023/A:1006398613605 10720205

[B33] Coons SW Pearl DK Mitosis identification in diffuse gliomas: implications for tumor grading Cancer 1998 15;82 8 1550 1555 10.1002/(SICI)1097-0142(19980415)82:8<1550::AID-CNCR17>3.0.CO;2-3 9554533

[B34] Sarafian TA Verity MA Vinters HV Shih CC Shi L Ji XD Dong L Shau H Differential expression of peroxiredoxin subtypes in human brain cell types J Neurosci Res 1999 15;56 2 206 212 10494109

[B35] Lee TH Kim SU Yu SL Kim SH Park do S Moon HB Dho SH Kwon KS Kwon HJ Han YH Jeong S Kang SW Shin HS Lee KK Rhee SG Yu DY Peroxiredoxin II is essential for sustaining life span of erythocytes in mice Blood 2003 15; 101 12 5033 5038 10.1182/blood-2002-08-2548 12586629

[B36] Hattori F Murayama N Noshita T Oikawa S Mitochondrial peroxiredoxin-3 protects hippocampal neurons from excitotoxic injury in vivo J Neurochem 2003 86 4 860 868 10.1046/j.1471-4159.2003.01918.x 12887684

[B37] Chang TS Cho CS Park S Yu S Kang SW Rhee SG Peroxiredoxin III, a mitochondrion-specific peroxidase, regulates apoptotic signaling by mitochondria J Biol Chem 2004 1;279 40 41975 41984 10.1074/jbc.M407707200 15280382

[B38] Plaisant F Clippe A Stricht D Vander Knoops B Gressens P Recombinant peroxiredoxin 5 protects against excitotocic brain lesions in newborn mice Free Radic Biol Med 2003 1;34 7 862 872 10.1016/S0891-5849(02)01440-5 12654475

[B39] Fujii T Fujii J Taniguchi N Augmented expression of peroxiredoxin VI in rat lung and kidney after birth implies an antioxidative role Eur J Biochem 2001 268 2 218 225 10.1046/j.1432-1033.2001.01843.x 11168354

[B40] Odreman F Vindigni M Gonzales ML Niccolini B Candiano G Zanotti B Skrap M Pizzolitto S Stanta G Vindigni A Proteomic studies on low- and high-grade human brain astrocytomas J Proteome Res 2004 4 3 698 708 10.1021/pr0498180 15952716

